# Efficacy and safety of sinomenine for diabetic kidney diseases: A meta-analysis

**DOI:** 10.1097/MD.0000000000036779

**Published:** 2023-12-29

**Authors:** Ying-Jie Zhang, Zong-Jie Shang, Mei Zheng, Ran Shi

**Affiliations:** a Institute of Traditional Chinese Medicine, Weifang Medical University, Weifang, China; b Department of Nephrology, Puyang Hospital of Traditional Chinese Medicine, Puyang, China; c Rehabilitation Medicine Department, The First Affiliated Hospital of Shandong First Medical University, Jinan, China.

**Keywords:** diabetic kidney disease, herbal medicine, meta-analysis, network pharmacology, sinomenine

## Abstract

**Background:**

In traditional Chinese medicine, Sinomenii Caulis contains Sinomenine (SIN), one of the major active ingredients. According to some studies, SIN can reduce proteinuria and provides clinical effectiveness rates in diabetic kidney disease (DKD) patients, however, the evidence is not strong and mechanisms of action are unclear. The efficacy and safety of SIN in treating DKD were evaluated by meta-analysis, and the potential mechanism of SIN therapy for DKD was initially explored by network pharmacology.

**Methods:**

PubMed, Cochrane Library, Embase, Web of Science, CNKI, Wanfang, VIP, and SinoMed databases were comprehensively searched until March 28, 2022. Randomized controlled trials on DKD treated with SIN were selected. The main results were clinical effective rate and the secondary results were the decrease in 24-hour urine total protein (24-hour UTP), serum creatinine, adverse reactions, etc. Drug combinations and disease stages were analyzed in subgroups. Sensitivity analysis was performed for 24-hour UTP. The potential target genes and pathways of SIN in treating DKD were studied using protein-protein interactions, gene ontology, and the Kyoto Genome Encyclopedia and Genomes enrichment analysis.

**Results:**

The meta-analysis included 7 randomized controlled trials. SIN treatment had a higher clinical effectiveness rate than conventional treatment (relative risk = 1.53, 95% confidence interval [1.30; 1.80], Z = 5.14, *P* < .0001); the decrease in 24-hour UTP, treatment group was higher than control group (standardized mean difference = −1.12, 95% confidence interval [−1.71; −0.52], Z = −3.69, *P* = .0002); In the experimental group, adverse reactions were more common than in the control group. SIN mainly affected 5 target genes, NFκB-1, TNF, interleukin 6, interleukin 1β and signal transducer and activator of transcription 3, and IL-17, AGE-RAGE signaling pathways, lipids, and atherosclerosis were all controlled to achieve therapeutic effects.

**Conclusion:**

SIN is an effective and safe drug for treating DKD, enhancing clinical efficacy, and reducing proteinuria. The main potential mechanism is anti-inflammatory.

## 1. Introduction

Diabetic kidney disease (DKD) is a microvascular complication of diabetes mellitus (DM) that is the main cause of end-stage renal disease (ESRD). Globally, approximately 80% of patients with ESRD develop DM, hypertension, or both. The risk of developing ESRD in diabetics is 10 times that of non-diabetics.^[1]^ According to the International Diabetes Federation, there will be 140.9 million Chinese who will have diabetes in 2021 and 536.6 million worldwide. By 2045, 783.2 million people worldwide will have diabetes,^[2]^ of which up to 45% will be affected by DKD.^[3]^ In developed countries, about 40% of patients face dialysis.^[4]^ DKD causes a great deal of socioeconomic and public health burden, thus, finding effective ways to prevent and treat it is crucial.

DKD is primarily treated by controlling blood glucose, blood pressure, and lipid levels. Clinically, angiotensin-converting enzyme inhibitors and (angiotensin receptor blockers) are considered effective in treating DKD.^[5,6]^ Inflammation and renal fibrosis are difficult to prevent with these drugs.^[7,8]^ In addition, it is clinically found that sodium-glucose cotransporter 2 inhibitor helps in controlling the progression of DM to DKD. However, it is still in the stage of clinical trials, and its effectiveness and safety are unknown.^[9]^ It is therefore essential to identify effective and safe medications to delay DKD development.

**Figure 1.**
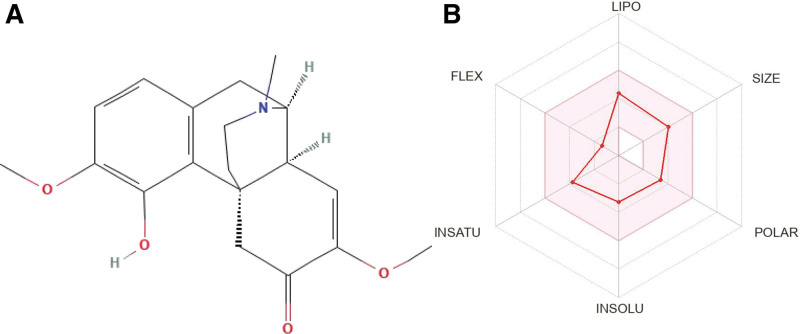
Basic information and network pharmacology analysis of SIN. Retrieved September 28, 2022 from https://pubchem.ncbi.nlm.nih.gov/ and http://www.swissadme.ch/. (A) 2D structure of SIN, (B) the physicochemical properties of SIN. SIN = Sinomenine.

Recently, the therapeutic effects of traditional Chinese medicine on DKD have attracted increasing attention from researchers and clinicians.^[7]^ In Chinese guidelines and consensus on diagnosing and treating DKD, various traditional Chinese medicine (TCM) monomers and TCM compound preparation are recommended for treating DKD.^[10–12]^ Sinomenii Caulis (SC) is the dried stem of *Sinomenium Acutum (Thunb.) Rehder & EHWilson.*^[13]^ It is a traditional Chinese herbal medicine, first recorded in The Tujing Herb in 1061 AD, that dispels rheumatism, dredges meridians and collaterals, and promoting urination.^[14]^ It is widely used in TCM prescriptions to treat DKD.^[15–17]^ Sinomenine (SIN) was isolated from SC by Ishiwari in the 1930s^[18]^ (Fig. 1A and B) and is considered a quality marker of SC in the Chinese, Japanese, Korean, and European pharmacopeia.^[14,19–21]^ The SIN content in the Chinese Pharmacopoeia should not be < 0.50%,^[14]^ which means that at least 500 mg of SIN can be found in 1 kg of SC. Some studies have clarified that SIN has anti-inflammatory,^[22]^ antitumor,^[23]^ analgesic,^[24]^ immune regulatory,^[25]^ diuretic,^[26]^ blood circulatory^[27]^ and other pharmacological effects. Animal studies have shown that SIN enhances renal capillary blood flow, reduces proteinuria excretion, and protects against renal injury. The mechanism is mainly related to mediating immune inflammatory reactions, such as inhibiting tumor necrosis factor (TNF)-α and nuclear factor-κB inflammatory factors and inhibiting CD4 + T-cell proliferation.^[28–30]^

SIN was developed by Zhengqing Fengtongning.^[31]^ There have been a number of clinical studies showing SIN can improve clinical efficacy, reduce proteinuria, and delay renal function in patients with DKD. However, these studies had small sample sizes and were single-center clinical trials with insufficient evidence of evidence-based medicine, but these results are inconsistent. In addition, the mechanism underlying SIN intervention in DKD remains unclear. Therefore, This study presents an initial meta-analysis of the safety and efficacy of SIN for treating DKD and a preliminary network pharmacology analysis on the potential mechanism of SIN to provide a clinical reference for treating DKD and mechanism research.

## 2. Materials and methods

The meta-analysis part of this study strictly followed the PRISMA extension statement for systematic reviews and meta-analyses.^[32]^ Here is a complete list of Preferred Reporting Items for Systematic Reviews and Meta-analyses in Supplementary Material, and the study was registered with PROSPERO (CRD:42022321168). Ethical approval is not necessary for this review study.

### 2.1. Inclusion and exclusion criteria

#### 2.1.1. Type of study.

Randomized controlled trials (RCTs).

#### 2.1.2. Object of study.

Patients diagnosed with DKD with staging criteria based on Mogensen et al^[33]^ Age, sex, disease course, and disease stage were not restricted in this study.

Intervention measures: Diabetes health education, blood glucose control, blood pressure lowering, blood lipid adjustment, and symptomatic treatment were provided to the control group. ACEI or ARB can be used to lower blood pressure without dose limitation. In addition, treatment group members received SIN preparation based on the control group, and intervention treatment was unlimited in duration.

#### 2.1.3. Outcome indicators.

Primary outcomes: clinical efficacy rate. Secondary outcomes: 24-hour urine total protein (24-hour UTP); serum creatinine (SCr); Glycosylated hemoglobin A1c (HbA1c); C-reactive protein (CRP); Adverse reactions (ADR).

#### 2.1.4. Exclusion criteria.

The full article was unavailable; no data were available for the article; republished studies only chose the latest and most comprehensive data, and interventions included other traditional Chinese medicine.

### 2.2. Literature retrieval strategy

Using PubMed, Embase, The Cochrane Library, and Web of Science, we searched 4 English databases as well as 4 Chinese databases [Chinese journal full text database (CNKI), Wanfang Digital Periodical Full Text Database, Chinese Scientific Journal Database (VIP), and China Biomedical Documentation Service System (SinoMed)] for RCTs on SIN for treating DKD. The retrieval time ranged from the establishment of the database to March 28, 2022, and the publications were unlimited. In addition, 3 clinical trial registration platforms (ClinicalTrials.gov, WHO International Clinical Trials Registry Platform (WHO ICTRP) (https://www.who.int/), and the Chinese Clinical Trial Register (Chinese CTR) (https://www.chictr.org.cn/) were searched to identify unpublished or ongoing trials. The retrieved words included “diabetic nephropathy”, “diabetic kidney disease”, “glomerulosclerosis”, “diabetes”, “sinomenine”, “cucoline hydrochloride”, and “Zhengqing Fengtongning”. Then, the Medical Subject Headings terms and keywords of these words were searched in the full text. There was no language, document type, or publication status restrictions.

### 2.3. Screening of literature and extraction of data

YingJie Zhang and Zong-Jie Shang independently reviewed the literature, extracted data, and checked data against 1 another. In cases of disagreement, relevant experts were consulted to help determine.We read the title and abstract of the literature first to exclude irrelevant literature, then the full text to determine whether to include it. Finally, using Microsoft Excel (Microsoft Corporation, Redmond, WA), the data were extracted. The main contents of the extracted data were as follows: the basic information of the study included title, first author, journal, publication time, research area, etc; detailed information on the baseline characteristics of the study subjects, such as the number of samples, age, gender, and disease status of each group; the specific measures and follow-up period of the intervention; the key elements of bias risk assessment; and the outcomes and measurement data.

### 2.4. Quality evaluation and statistical analysis

To assess bias risk in randomized trials, ROB2, a Cochrane tool, was used.^[34]^ Various aspects of the study were analyzed, including randomization, deviations from the intended intervention, missing outcome data, measurement of the outcome, and report selection. These 5 items were evaluated as low risk, some concerns, and high risk. Two evaluators conducted the bias risk assessment independently, and the results were compared. If there were any differences, a third evaluator made the decision.

This study used the meta-packages in *R* × 64 4.1.2 For statistical analysis, use the (Foundation for Statistical Computing, Vienna, Austria). In order to analyze continuous data, the Mean Difference (MD) or standardized mean difference (SMD) was used. Relative risk was applied to dichotomous data. The confidence interval (CI) was set to 95%.Using the Q test, the heterogeneity of the studies was examined. When *P* > .1 and *I*^2^ < 50%, no statistical heterogeneity was observed, and analyses were conducted using the common effect model. In contrast, it has statistical heterogeneity; therefore, to deal with heterogeneity among studies, we should adopt a random-effects model and use subgroup analysis. This result was displayed using a forest map or descriptive analysis.

### 2.5. Research on potential mechanisms

PubChem was used to derive SIN’s 2D structure (Fig. 1A). The Target Prediction platform was used to identify potential target genes (http://www.swisstargetprediction.ch/) and the HERB database (http://herb.ac.cn/). The GeneCards database (https://www.genecards.org/) was used to identify genes related to DKD. The intersection of disease and drug genes produced the potential genes. Then, the network map of “SIN -Gene-DK” was drawn using Cytoscape3.8.0 (http://www.cytoscape.org/) Based on the STRING database (https://string-db.org/), a Protein-Protein Interaction (PPI) network was constructed of potential genes, and important genes were obtained by topology analysis using CytoNCA in Cytoscape.^[35]^

The ClusterProfiler package^[36]^ in R was used for gene ontology (GO) and Kyoto encyclopedia of genes and genomes (KEGG) enrichment analyses, and the threshold was set at *P* < .05. GO analysis can be used to obtain the results of biological processes (BP), cellular components, and molecular functions (MF). KEGG analysis can enrich the genes into action pathways. Five items were selected with the smallest q values in BP, cellular components, and MF, and 15 pathways with the smallest q values in KEGG. A relationship network with important genes was constructed using Cytoscape to identify key target genes.

## 3. Results

### 3.1. Literature screening process and results

**Figure 2.**
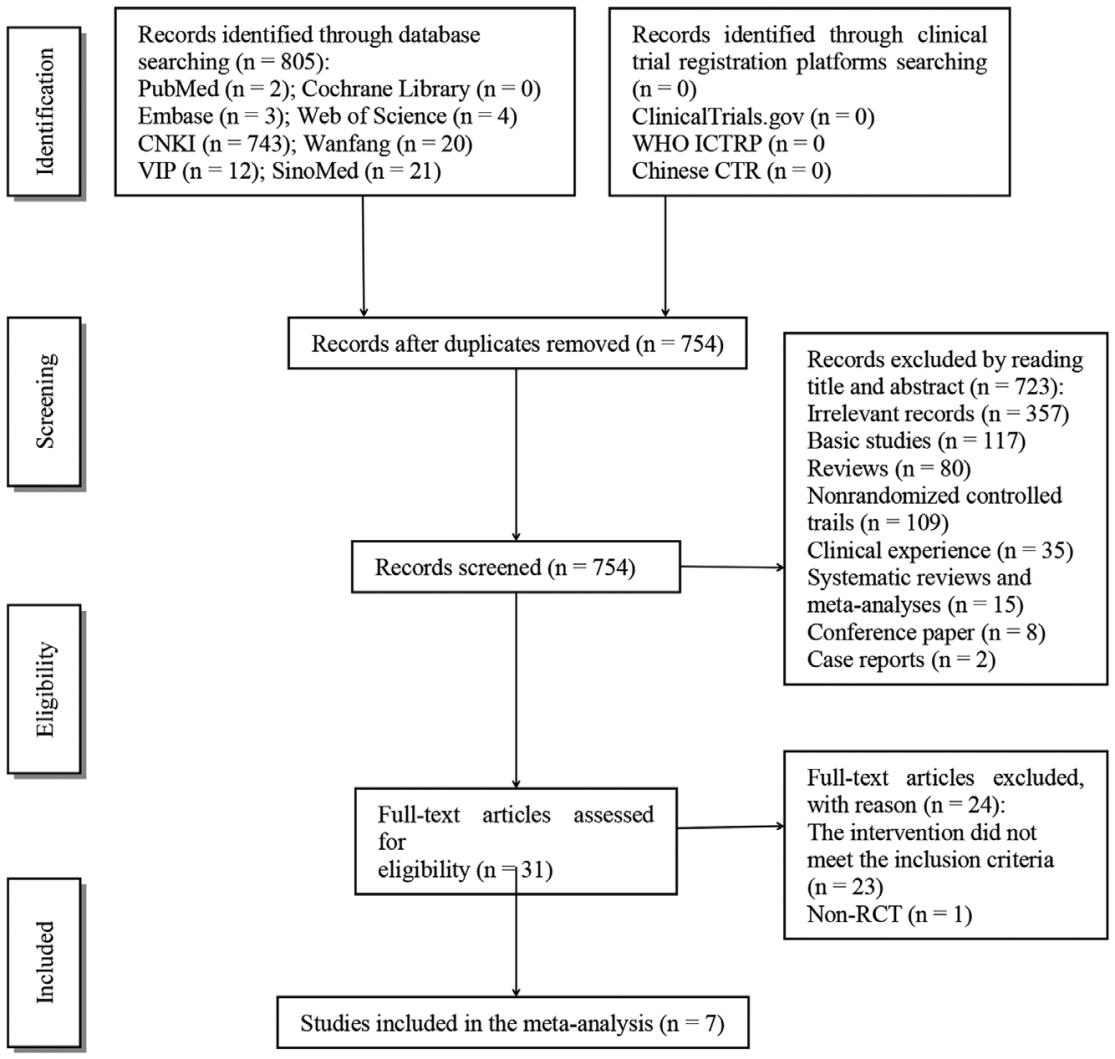
Literature screening flow diagram based on PRISMA.

There were initially 805 related studies identified, and 7 RCTs were finally included after layer-by-layer screening,^[37–43]^ with 452 patients. A description of the literature screening process and the results is provided in Figure 2.

### 3.2. Literature characteristics and quality evaluation

The 7 studies are described based on their basic characteristics in Table 1. In comparison to the control group, there was no significant difference in baseline data (*P* > .05). These 7 studies were from China and published between 2009 and 2020, with a minimum sample size of 40 cases, a maximum sample size of 108 cases, and 452 patients (267 males and 185 females). The treatment duration was 2, 8, or 12 weeks. Two hundred twenty-three patients received routine basic treatment in the control group. Among the group receiving treatment, 229 patients were treated with Zhengqing Fengtongning sustained-release tablets based on the control group. All included studies reported adverse reactions.

**Table 1:** Basic characteristics of included RCTs

Author	Stages	Age (yr)	Sample size (male/female)	Durations	Intervention methods		AR (T/C)	Outcomes
					T	C		
Gen Li 2018^[37]^	DKD 3	T:59.20 ± 4.96 C:57.16 ± 6.20	T:25 (20/5) C:25 (18/7)	8 wk	ZFSRT 120 mg Bid po + Irbesartan 150 mg Qd	Irbesartan 150 mg Qd	0/0	①③④
Chao Wang 2018^[38]^	DKD 4	T:34-67 C:32-65	T:36 (25/11) C:36 (26/10)	12 wk	ZFSRT 120 mg Bid po + Losartan Potassium 50 mg Qd	Losartan potassium 50 mg Qd	5/2	①②③
Rui Chen 2012^[39]^	DKD 4	T:44.7 ± 12.0 C:45.4 ± 11.5	T:38 (20/18) C:32 (15/17)	3 mo (12wk)	ZFSRT 120 mg Bid po + Valsartan capsules 80 mg Qd	Valsartan capsules 80 mg Qd	1/0	①②③④
Laimin Luo 2009^[40]^	DKD	36-67	T:20 C:20	2 wk	ZFSRT 120 mg Bid po	Supportive therapy	5/0	②③
Wen Long 2016^[41]^	DKD 3-4	T:57.50 ± 8.26 C:54.21 ± 9.35	T:24 (13/11) C:24 (14/10)	12 wks	ZFSRT 120 mg Bid po + Valsartan capsules 80 mg Qd	Valsartan capsules 80 mg Qd	0/0	①②④⑤
Jingyu Zhao 2012^[42]^	DKD 3	40-75	T:54 C:54	8 wk	ZFSRT 120 mg Bid po + Olmesartan Medoxomil 10 mg Qd	Olmesartan medoxomil 10mg Qd	8/0	②④
Fanxue Zeng 2020^[43]^	DKD 3	T:54.94 ± 8.38 C:52.13 ± 7.09	T:32 (18/14) C:32 (19/13)	8 wk	ZFSRT 120 mg Bid po + Candesartan cilexetil 4 mg-12 mg Qd	Candesartan cilexetil 4 mg-12 mg Qd	2/1	①②③④⑤

AR = adverse reactions, C = control group, DKD = diabetic kidney disease, po = per os, Qd = quaque die, RCTs = Randomized controlled trials, T = treatment group, ZFSRT = Zhengqing Fengtongning Sustained-Release Tablets.

**Figure 3.**
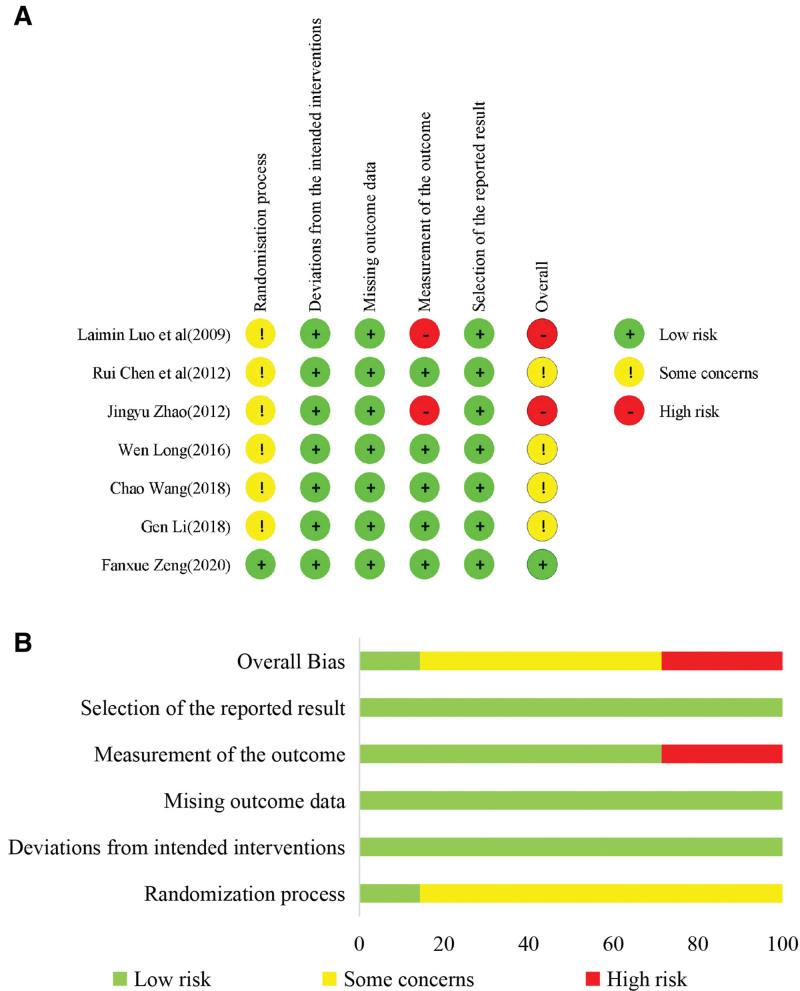
Quality evaluation results of included RCTs. (A) RCTs included in the risk of bias assessment, (B) inclusion of RCTs with varying risk of bias. RCTs = randomized controlled trials.

Based on the bias risk assessment, 1 study was found to be biased^[43]^ mentioned a random number method with a hidden distribution method for random allocation. Two studies^[38,41]^ mentioned that only random allocation was performed using a random number generator; 4 studies^[37,39,40,42]^ referred only to random assignment. Zeng^[43]^ reported the situation and causes of shedding, whereas the other studies did not report the situation of loss to follow-up. The outcome measurement methods of 2 studies^[40,42]^ may be inappropriate, and the data were not checked to determine whether they conformed to a normal distribution. The desired indicators have been reported previously. The evaluation results are shown in Figure 3.

### 3.3. Results of meta-analysis

#### 3.3.1. Effective rates.

**Figure 4.**
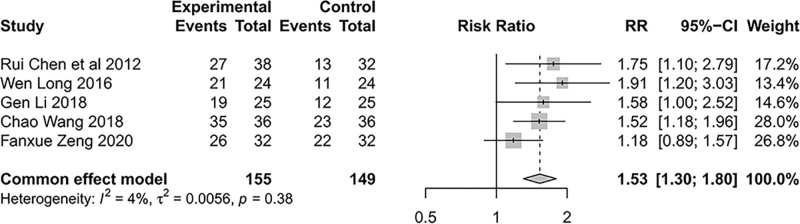
Forest plot of meta-analysis of clinical effective rate of SIN intervention on DKD. DKD = diabetic kidney diseas, SIN = Sinomenine.

Five studies^[37–39,41,43]^ reported the clinical efficacy, a set of efficacy criteria was developed based on the TCM guiding principles for clinical research of new drugs.^[44]^ The studies did not show significant heterogeneity(*I*^2^ = 4%, *P* = .38). The results obtained using a common effects model revealed that the treatment group had higher clinical effectiveness rates than the control group (relative risk = 1.53, 95% CI [1.30; 1.80], Z = 5.14, *P* < .0001) (Fig. 4).

#### 3.3.2. Proteinuria level.

**Figure 5.**
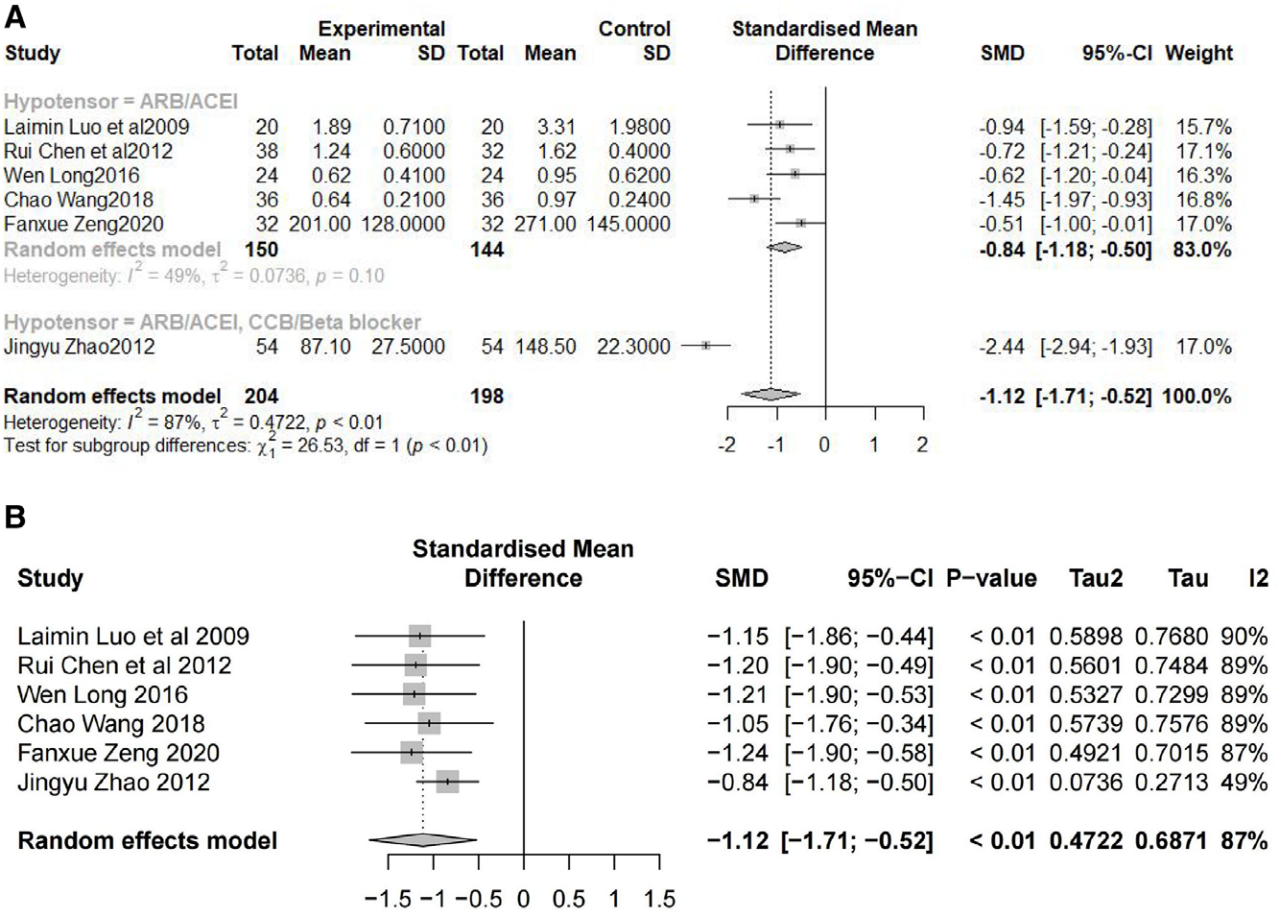
Proteinuria indexes of SIN intervention on DKD. (A) Forest plot of meta-analysis of 24-hour UTP, (B) sensitivity analysis of the meta-analysis of 24-hour UTP. 24-hour UTP = 24-hour urine total protein, DKD = diabetic kidney diseaseSIN = Sinomenine.

The effect of SIN on proteinuria was evaluated by 24-hour UTP. Six studies^[38–43]^ reported 24-hour UTP, treatment group: 92, control group: 92. Studies were heterogeneous (*I*^2^ = 87%, p0.01), and a random-effects model was used to analyze the data. The treatment group had significantly lower 24-hour UTP than the control group (SMD = −-1.12, 95% CI [−1.71; −0.52], Z = −3.69, *P* = .0002). Reading the original article revealed that Zhao^[42]^ used ARB combined with CCB/Beta blocker to control blood pressure. We conducted a subgroup analysis according to the use of hypotensors and found that the main source of heterogeneity was the difference in the use of hypotensors (χ_1_^2^ = 26.53, *P* < .01) (Fig. 5A). We then performed a sensitivity analysis and determined that the heterogeneity originated from the study by Zhao^[42]^ (Fig. 5B).

#### 3.3.3. Renal function indexes.

**Figure 6.**
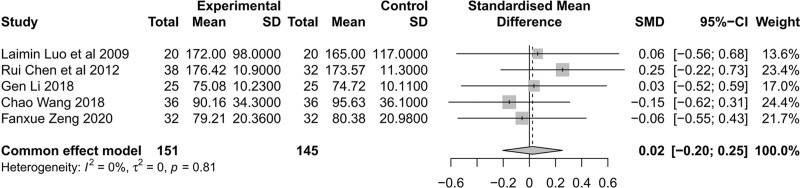
Forest plot of meta-analysis of serum creatinine of SIN intervention on DKD. DKD = diabetic kidney disease, SIN= Sinomenine.

Renal function was assessed using SCr. Five studies^[37–40,43]^ included 151 patients in the treatment group and 145 patients in the control group, and SCr levels were reported. There was no significant heterogeneity among the studies (*I*^2^ = 0%, *P* = .81), and SCr was not significantly different between the treatment and control groups based on the common effect model (SMD = 0.02, 95% CI [− 0.20; 0.25], Z = 0.21, *P* = .8340). The results showed that SIN had no adverse effects on renal function in patients with DKD and was relatively safe, which may be because of the short observation period (Fig. 6).

#### 3.3.4. Glycosylated hemoglobin A1c and C-reactive protein.

**Figure 7.**
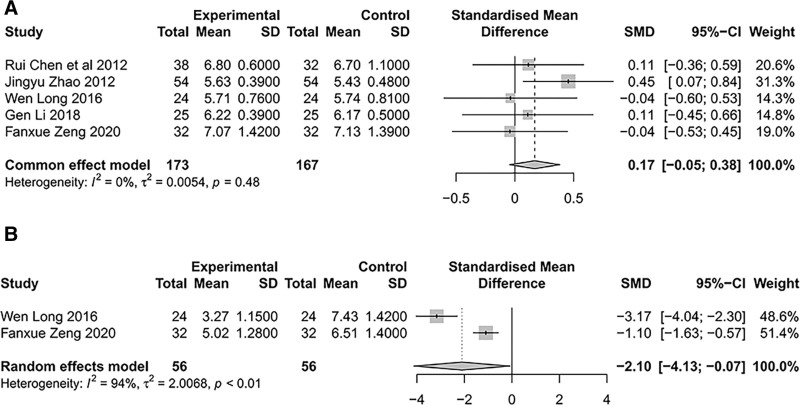
HbA1c and CRP of SIN intervention on DKD. (A) Meta-analysis of HbA1c forest plot, (B) meta-analysis of CRP forest plot. CRP = C-reactive protein, DKD = diabetic kidney disease, HbA1c = glycosylated hemoglobin A1c, SIN = Sinomenine.

HbA1c was reported in 5 studies,^[37,39,41–43]^ and among the studies, there was no heterogeneity (*I*^2^ = 0%, *P* = .48). HbA1c levels did not differ significantly between treatment and control groups based on the common effect model (SMD = 0.17, 95% CI [- 0.05; 0.38], Z = 1.54, *P* = .1224) (Fig. 7A). In patients with DKD, SIN did not adversely affect blood glucose levels.

CRP was reported in 2 studies^[41,43]^ with significant heterogeneity between studies (*I*^2^ = 94%, *P* < .01), and our analysis was based on a random-effects model. Compared to the control group, the treatment group had significantly lower CRP (SMD = −2.10, 95% CI [−4.13; −0.07], Z = −2.03, *P* = .0421). A literature analysis of disease stage, treatment duration, and ARB may be the sources of heterogeneity in the 2 studies. Long^[41]^ included CKD III–IV patients; the treatment duration was 12 weeks, and the treatment plan was to administer ARB based on qualified blood pressure control. Zeng^[43]^ included patients with CKD III; the treatment duration was 8 weeks, and the treatment plan was to use ARB for blood pressure control with different doses among patients (Fig. 7B). As a result of these results, CRP levels may be reduced in patients with DKD when SIN is administered.

#### 3.3.5. Adverse reaction.

Seven studies reported ADR, 2 of which did not occur, and the other 5 reported ADRs in 24 patients (Treatment group: 21; control group: 3). There were 16 patients in the treatment group with rash and pruritus, and 3 patients had mild gastrointestinal discomfort, which disappeared after tolerance or symptomatic treatment. Wang^[38]^ reported 4 cases of dizziness, fatigue, and palpitations groups treated and untreated, which may be related to Losartan Potassium Tablets. Zeng et al^[43]^ reported hypotension in a control group that improved after adjusting for antihypertensive drugs. None of the aforementioned factors affected the research progress. The results showed that the SIN was associated with fewer adverse reactions and was safe in patients with DKD.

### 3.4. Results of the mechanism analysis

**Figure 8.**
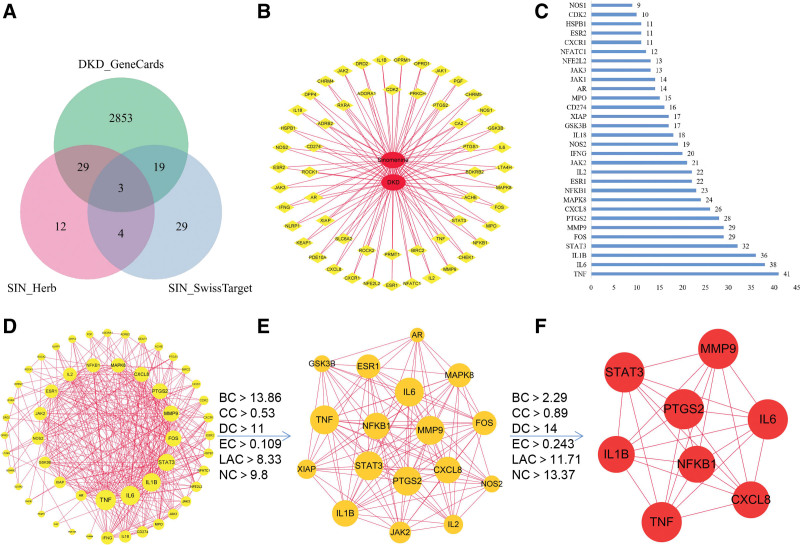
Network pharmacology analysis of SIN. (A) Venn diagram of target genes of SIN and DKD, and the intersection region are common genes, (B) the interaction network diagram of SIN - target - DKD was constructed by using common target gene, (C) top 30 target genes for degree in string PPI network, (D) diagram of a protein-protein interaction network, as the number of linker proteins increases, the size of the node increases, (E) networks obtained for the first analysis of CytoNCA, (F) this network was obtained by the second CytoNCA analysis, and the nodes in the network are Hub genes for SIN intervention on DKD. BC = betweenness centrality, CC = closeness centrality, DC = degree centrality, DKD = diabetic kidney disease, EC = eigenvector centrality, LAC = local average connectivity, NC = network centrality, PPI = protein-protein interaction, SIN = Sinomenine.

Fifty-five target genes of SIN were obtained from the Swiss target Prediction, 48 target genes of SIN were obtained from HERB, and 2904 target genes of DKD were obtained from the GeneCards database (Relevance Score ≥ 1). After the intersection, 55 common target genes of SIN and DKD were identified. These 55 genes were used to construct an interaction network between SIN and DKD, which may represent the potential action network of SIN in DKD (Fig. 8A and B). We used CytoNCA in Cytoscape to analyze the STRING PPI network and selected genes with betweenness centrality, closeness centrality (CC), degree centrality, eigenvector centrality, local average connectivity, and network centrality higher than their median for the 2 screenings. It was found that CXC chemokine ligand 8, Interleukin 1β (IL-1β), *NFκB-1*, matrix metalloproteinase 9, interleukin 6 (IL-6), TNF, prostaglandin-endoperoxide synthase 2 and signal transducer and activator of transcription 3 (STAT-3) may be the hub genes for SIN to treat DKD (Fig. 8C, D–F).

**Figure 9.**
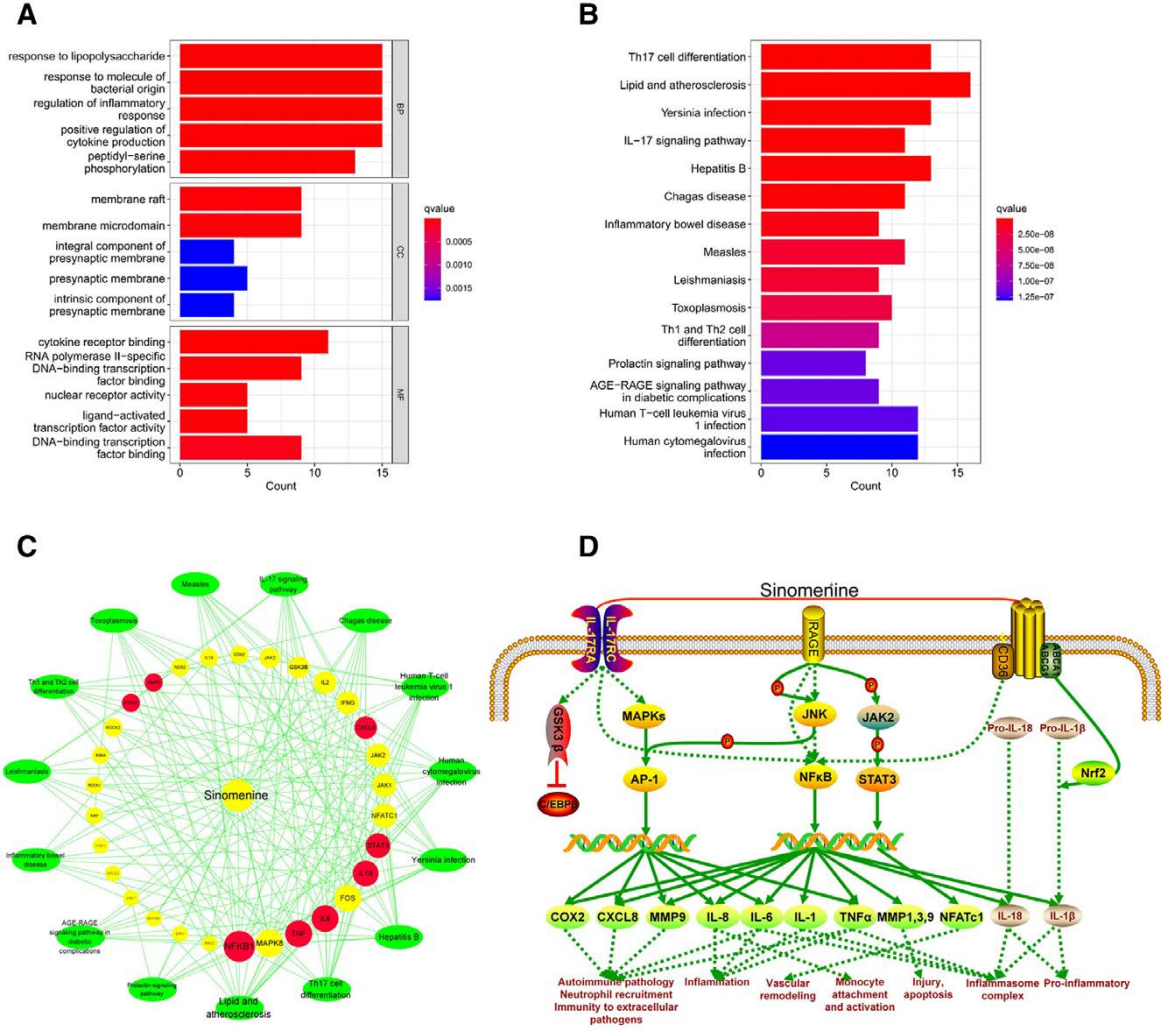
Potential mechanism analysis of SIN intervention in DKD. (A) Histograms of BP,CC and MF enrichment analysis for the five smallest q values in GO enrichment analysis, (B) histogram of pathway analysis with the 15 smallest q values in KEGG enrichment analysis, (C) SIN - target - pathway network diagram. Protein genes are represented by the inner circle nodes, and Hub genes are represented by the red nodes. The outer ring node represents the pathway, (D) an illustration of possible molecular mechanisms of SIN used to treat DKD. (SIN plays a role in treating DKD by interfering with the targeted genes in the diagram, and the mode of action is at the bottom of the diagram). BP = biological processes, CC = cellular components, DKD = diabetic kidney disease, GO = gene ontology, KEGG = Kyoto encyclopedia of genes and genomes, MF = molecular functions, SIN = Sinomenine.

By analyzing GO enrichment results, 1742 results were obtained (q value < 0.05), of which BP had 1638 items, majorly involved in lipopolysaccharide response, inflammation regulation, and cytokine production regulation. The CC had 26 items, mainly involving membrane rafts, membrane microdomains, and integral components of the presynaptic membrane. MF comprised 78 items, it primarily involves the binding of cytokines to receptors, the activity of nuclear receptors, and the activation of transcription factors via ligands. The top 5 items (betweenness centrality, CC, and MF) were selected according to the q value to draw histograms (Fig. 9A). KEGG enrichment analysis revealed 115 signaling pathways (q value < 0.05). It mainly includes Th17 cell differentiation, lipid accumulation, atherosclerosis, and the IL-17 signaling pathway. The first 15 pathways with q values were shown in Figure 9B. The interaction network between the first 15 informational pathways and target genes was constructed using Cytoscape, and the core target genes were labeled. It was found that NFκB-1, TNF, IL-6, IL-1β, and STAT-3 might be the key target genes for SIN to treat DKD. In addition, Th17 cell differentiation, lipids, and atherosclerosis may be key pathways for SIN in treating DKD (Fig. 9C).

## 4. Discussion

### 4.1. Clinical effectiveness analysis

DKD can reduce the life expectancy of patients by 16.9 years,^[45]^ a heavy burden is placed on the families of the patients and society as a whole. The mechanism of DM progression into DKD is generally believed that many advanced glycations end products (AGEs) can be produced in the body of DM patients under a high glucose environment.^[46]^ AGEs can not only activate the RAAS to cause increased glomerular filtration pressure, but also lead to excessive release of reactive oxygen species and inflammatory neurotransmitters, thereby damaging podocytes and leading to a series of pathological changes, such as glomerular basement membrane hyperplasia, glomerular sclerosis, inflammation, and fibrosis in the renal tubule interstitium. These changes also cause increased urinary protein levels, elevated blood pressure, and progressive renal insufficiency in DKD.^[47]^ The traditional treatment, however, mainly focuses on controlling blood glucose, blood pressure, blood lipids, and proteinuria, which cannot effectively combat inflammation and fibrosis.

SIN is the main active component of the SC. It has been found that SIN can reduce the renal inflammatory response and delay renal cell sclerosis and interstitial fibrosis by down-regulating inflammatory factors, inhibiting immune-mediated inflammatory responses, and reducing AGEs.^[48]^ This study included 7 articles, 5 of which assessed the clinical effectiveness of SIN in treating DKD, 6 articles evaluated the efficacy of proteinuria from 24-hour UTP, and 2 articles evaluated CRP, with statistical differences in all indicators. In addition, renal function was evaluated in 5 articles and blood glucose was assessed in 5 articles, with no significant difference between the 2 indices. SIN based on conventional treatment could improve clinical efficiency compared to conventional treatment of DKD, reduce the level of proteinuria, improve the level of inflammation, and have no significant effect on renal function or blood glucose levels. Evaluation of adverse reactions was conducted in all articles and was statistically different, mainly for rash and pruritus. No severe adverse reactions or effects were observed during the treatment.

### 4.2. Suggestions for clinical research

SIN combined with conventional treatment of DKD is superior to conventional treatment alone, according to the results of this study, without serious adverse reactions. Therefore, the clinical treatment of DKD can be considered in the future by combining SIN with conventional treatment, which can increase the treatment benefit. In addition, it is suggested that the drug administration scheme should be reasonably planned according to the individual situation of patients on clinical medication to reduce the adverse reactions, and follow-up should be paid attention to during treatment to observe the long-term efficacy of drugs.

This study strictly controlled for co-intervention measures, but there were differences between treatment, and drug application of basic treatment in each study, and some outcomes were still heterogeneous. Therefore, this study requires a more detailed design, more samples, stricter operation, and large-scale, multi-center RCT support. Clinical research should focus on improving the quality of methodology, clarifying the details of random assignment, assigning hidden and blind objects, standardizing clinical reports, and suppressing bias, to provide evidence-based evidence for follow-up clinical research.

### 4.3. Potential mechanism analysis

Based on the results of this meta-analysis, using network pharmacology, we investigated potential mechanisms of SIN treatment for DKD. The results showed that SIN affected DKD cells through 55 target genes, and many pathways were identified by GO and KEGG enrichment analyses. Among them, the AGE-RAGE signaling pathway in diabetic complications, IL-17 signaling pathway, lipids, and atherosclerosis of KEGG were closely related to SIN treatment of DKD. Furthermore, the target genes enriched in the KEGG pathway were crossed with the core target genes of PPI, and TNF,^[49]^ IL-6,^[50]^ IL-1β,^[51]^ *NFκB-1*,^[52]^ and STAT-3^[53]^ were identified as hub genes that are closely related to the inflammatory response in DKD.

The inflammatory response is one of the main mechanisms underlying DKD. It has been found that the course of DKD is positively correlated with inflammatory mediators in plasma^[54,55]^ and closely related to the excretion of urinary albumin.^[56,57]^ Activation of numerous transcription factors and kinases promotes the production of pro-inflammatory trend factors, cytokines, and adhesion factors, and accumulate in the renal tissue, thereby inducing renal injury.^[58]^ Meta-analysis showed that 24-hour UTP, ALB, and β2-MG were significantly different. Simultaneously, it was found that SIN could down-regulate the levels of TNF-α, IL-6, and IL-1β in transplanted kidney tissue of rats and produce an immunosuppressive effect,^[29]^ and reduce the levels of IL-6 and IL-18 in serum of DKD rats to inhibit the inflammatory response, to reduce the 24-hour UTP of DKD rats and alleviate the pathological changes of kidney.^[59]^ It was also found that SIN could reduce the infiltration of CD3 + and CD68 + positive cells in DM rats, reduce the levels of TNF-α, IL-1, and IL-6, inactivate nuclear factor-κB, and block cytokine-mediated immune inflammatory response^[60]^; It can also reduce the level of The Kelch-like ECH-associated protein 1 (keap1) and activate Nuclear factor-erythroid 2-related factor (Nrf2) signaling protein expression to inhibit inflammation and oxidative stress.^[61]^ In addition, SIN can regulate the JAK2/STAT-3/SOCS1 signaling pathway in DKD rats, reduce the levels of p-JAK2 and p-SRAT3, and reduce the expression of the downstream inflammatory factor IL-6, thereby this inhibits oxidative stress as well as reduces apoptosis and fibrosis in renal cells.^[62]^ These results are consistent with the inflammatory signal transduction pathways identified in this study, such as the AGE-RAGE signaling pathway in diabetic complications, the IL-17 signaling pathway, lipids, and atherosclerosis. SIN inhibited inflammation by interfering with related target genes, thereby reducing the level of proteinuria and alleviating the curative effect on DKD (Fig. 9D). The articles included in this study did not comprehensively report inflammatory indicators; only 2 articles compared inflammatory indicators with CRP, and the analysis found that there were statistical differences, but there was high heterogeneity. therefore, more clinical evidence is needed to support this.

### 4.4. Shortcomings and limitations

This study included 7 low-quality articles in total, of which only one article described the randomization process in detail, and 6 articles reported randomization. Therefore, the outcome strategy may be biased, and these 2 articles may not be appropriate for the main outcome measurement analysis method. These articles were single-center studies with a total sample size below 120, and the sample size estimation was not introduced in detail. Large-scale, multi-center, randomized controlled trials are lacking. Based on the current evidence, although the overall efficacy of the treatment group was higher than that of the control group limited by the number and overall level of included studies, the aforementioned conclusions need further verification by subsequent high-quality RCTs. Network pharmacology is a preliminary technique based on virtual strategies. Its advantage is that it improves the research direction of relevant molecules. Its disadvantage is that some false-positive results may be obtained; therefore, this needs to be further verified by subsequent in vivo and in vitro experiments. In addition, network pharmacology ignores the influence of ingredient content on the experimental results, and the relationship between ingredient content and curative effects should be considered in future studies.

## 5. Conclusion

This study evaluated the clinical efficacy of SIN in treating DKD based on a meta-analysis and network pharmacology. Treatment group clinical effectiveness, 24-hour UTP, and CRP were superior to control group values. A total of 55 target genes and 115 pathways were obtained from an online database that was closely related to the treatment of DKD, atherosclerosis, lipids, and AGE-RAGE signaling pathways were primarily involved in diabetic complications. Based on existing evidence, using SIN based on conventional treatment can significantly improve the clinical efficacy of DKD and reduce proteinuria and inflammation. However, the overall level of evidence is low and large-scale, multi-center RCTs are still needed for further support. The treatment of DKD by SIN is a complete result of multi-target and multi-pathway interactions, which mainly treat DKD by intervening in inflammatory response pathways.

## Acknowledgments

The English language editing was done by Editage (www.editage.cn), and this research has no conflict of interest with Editage.

## Author contributions

**Conceptualization:** Ying-Jie Zhang, Ran Shi.

**Data curation:** Ying-Jie Zhang, Zong-Jie Shang.

**Formal analysis:** Ying-Jie Zhang, Zong-Jie Shang.

**Funding acquisition:** Ying-Jie Zhang.

**Investigation:** Ying-Jie Zhang.

**Methodology:** Ying-Jie Zhang, Zong-Jie Shang, Mei Zheng, Ran Shi.

**Project administration:** Ying-Jie Zhang, Zong-Jie Shang.

**Software:** Ying-Jie Zhang, Zong-Jie Shang, Mei Zheng.

**Supervision:** Ran Shi.

**Visualization:** Zhang Yingjie.

**Writing – original draft:** Zhang Yingjie.

**Writing – review & editing:** Zhang Yingjie.
